# Differential Expression of Cytochrome P450 Enzymes in Normal and Tumor Tissues from Childhood Rhabdomyosarcoma

**DOI:** 10.1371/journal.pone.0093261

**Published:** 2014-04-03

**Authors:** Dora Molina-Ortiz, Rafael Camacho-Carranza, José Francisco González-Zamora, Jaime Shalkow-Kalincovstein, Rocío Cárdenas-Cardós, Rosario Ností-Palacios, Araceli Vences-Mejía

**Affiliations:** 1 Instituto Nacional de Pediatría, Mexico City, Mexico; 2 Instituto de Investigaciones Biomédicas, Universidad Nacional Autónoma de México, Mexico City, Mexico; 3 Centro Nacional para la Prevención y el Tratamiento del Cáncer en la Infancia y la Adolescencia (CeNSIA), Mexico City, Mexico; The Chinese University of Hong Kong, Hong Kong

## Abstract

Intratumoral expression of genes encoding Cytochrome P450 enzymes (CYP) might play a critical role not only in cancer development but also in the metabolism of anticancer drugs. The purpose of this study was to compare the mRNA expression patterns of seven representative CYPs in paired tumor and normal tissue of child patients with rabdomyosarcoma (RMS). Using real time quantitative RT-PCR, the gene expression pattern of CYP1A1, CYP1A2, CYP1B1, CYP2E1, CYP2W1, CYP3A4, and CYP3A5 were analyzed in tumor and adjacent non-tumor tissues from 13 child RMS patients. Protein concentration of CYPs was determined using Western blot. The expression levels were tested for correlation with the clinical and pathological data of the patients. Our data showed that the expression levels of CYP1A1 and CYP1A2 were negligible. Elevated expression of CYP1B1 mRNA and protein was detected in most RMS tumors and adjacent normal tissues. Most cancerous samples exhibit higher levels of both CYP3A4 and CYP3A5 compared with normal tissue samples. Expression of CYP2E1 mRNA was found to be significantly higher in tumor tissue, however no relation was found with protein levels. CYP2W1 mRNA and/or protein are mainly expressed in tumors. In conclusion, we defined the CYP gene expression profile in tumor and paired normal tissue of child patients with RMS. The overexpression of CYP2W1, CYP3A4 and CYP3A5 in tumor tissues suggests that they may be involved in RMS chemoresistance; furthermore, they may be exploited for the localized activation of anticancer prodrugs.

## Introduction

Rhabdomyosarcoma (RMS) is the most common soft tissue sarcoma in children. This sarcoma has presumed skeletal muscle origins, due to its myogenic phenotype [1]. The most common site in which RMS occurs is in the head and neck structures (∼40%), genito-urinary tract (∼25%), and extremities (∼20%) [2]. However, the etiology and pathogenesis of RMS are still poorly understood, although the genetics and environmental factors appear to each play a role in the neoplastic transformation [3], [4]. Embryonal RMS and alveolar RMS are the two major RMS subtypes that exhibit distinct morphology and genetic make-up. The patient’s prognosis is dependent on the localization of the primary lesion, stage of disease, age at diagnosis and histological subtype [5]. Chemotherapy is one of the three most common treatment modalities in RMS, but often, the resistance of cancer cells to drugs limits its efficacy [6], [7].

Over-expression of the drug metabolizing enzymes knows as Cytochrome P450 (CYP) is considered one of the major mechanisms of chemoresistance in solid tumors [8]. CYP is a multigene family of constitutive and inducible enzymes involved in the metabolism of endogenous and exogenous compounds, such as drugs, environmental pollutants and dietary components [9]. However, CYP enzymes not only function in the detoxification of xenobióticos but may also be involved in the activation of potential (pro-)carcinogens and toxicants [10]. CYP families 1, 2, and 3, which are the main CYP families participating in the metabolism of xenobiotics, are highly expressed in the liver; however they are also expressed in a range of extrahepatic tissues (e.g. intestine, brain, kidney, placenta, lung, adrenal gland, pancreas, skin, mammary gland, uterus, ovary, testes and prostate) [11]. Furthermore, it has been suggested that the local expression of CYPs in tumors is important for the management of cancer because CYPs expressed in tumors may be involved in the activation and/or inactivation of chemotherapeutic drugs [12]. Consequently, local CYP enzymes might also play a critical role not only in the development but also in the treatment of RMS. However, to the best of our knowledge, the expression of specific forms of CYP genes in RMS has not been previously described. Thus, the aim of the present study was to determine the mRNA expression pattern of seven representative CYPs (e.g., CYP1A1, CYP1A2, CYP1B1, CYP2E1, CYP2W1, CYP3A4, and CYP3A5) in paired tumor and normal tissue of childhood patients with RMS. Our findings showed that there are differences in the specific CYP mRNA expression between paired tumors and normal tissues. These altered CYP expression levels might also play important roles both in the etiology of RMS and as determinants of the success of RMS patients’ therapy.

## Materials and Methods

### Ethics

This study was approved by the research and ethics committees of the Instituto Nacional de Pediatría and was performed according to the Declaration of Helsinki. The patient’s parents or guardians and children older than 12 years provided their informed written consent to participate in this study.

### Patients and Samples

Biopsies of the tumor and paired normal tissue were obtained from 13 patients with RMS who were diagnosed prospectively and recruited at the Instituto Nacional de Pediatría (Mexico City, Mexico) during the period of 2010 to 2012. None of the patients had received radiotherapy or chemotherapy prior to the surgery. All samples were further processed immediately after the surgical biopsy at the time of diagnosis. The tumor samples were obtained from the malign mass. Adjacent normal tissues were obtained from the tissues, which showed no sign of cancer by visual inspection, that were located within 2 to 5 cm of the boundary of the cancer site. Histological classification of the RMS tumor samples, as well as confirmation of non-tumor cells in the normal specimens, were made according to standard diagnostic procedures and confirmed by pathologists. Demographic and clinical characteristics of the 13-patient study population are shown in Table 1. After procurement the samples were immediately submerged in RNAlater solution (Ambion, Courtaboeuf, France) to avoid RNA degradation, stored at 4°C for 24 h and subsequently at −80°C for further analyses.

**Table 1:** Demographic and clinical features of patients involved in the study.

Patient #	Age (years, months)	Gender	Primary sites*	Histology
1	0,5	F	H&N	Alveolar
2	4,7	F	H&N	
3	6,4	M	H&N	
4	7,1	M	EX	
5	8,7	F	EX	
6	11,1	M	EX	
7	11,9	M	H&N	
8	12,1	M	H&N	
9	14,6	M	H&N	
10	3,8	M	H&N	Embryonal
11	4,0	M	H&N	
12	6,7	F	GU	
13	12,1	M	H&N	

*Primary sites: EX = Extremities; GU = Genitourinary; H&N = Head and Neck.

### Isolation of Total RNA

Total RNA was isolated from paired tumor and normal tissue samples of each patient using the Trizol (Invitrogen Life Technologies Carlsbad, CA, USA) extraction method according to the manufacturer’s instructions. The integrity of the RNA was verified by visual inspection of the ethidium bromide stained 28S and 18S ribosomal RNA bands after agarose gel electrophoresis. The sample purity was ensured with an OD_260_/OD_280_ ratio >1.8, as measured using a NanoDrop ND-1000 spectrophotometer (NanoDrop Technologies, Wilmington, DE).

### Reverse Transcription and qPCR

A two-step protocol was employed. For cDNA synthesis 2 μg of each total RNA sample was reverse-transcribed in a final reaction volume of 100 μL containing 1×reverse transcription-PCR buffer, 5.5 mM/L MgCl2, 500 μM/L each of deoxynucleotide triphosphate, 2.5 μM/L random hexamers, 0.4 units/μL RNase inhibitor, and 1.25 units/μL multiScribe reverse transcriptase (Applied Biosystem, Rotkreuz, Switzerland). The mixture was incubated at 25°C for 10 min, 48°C for 30 min, and 95°C for 5 min.

The following probes were obtained for quantitative RT-PCR (qRT-PCR) analysis from Applied Biosystems: CYP1A1 (Hs00153120), CYP1A2 (Hs01070374), CYP1B1 (Hs00164383), CYP2E1 (Hs00559370), CYP2W1 (Hs00908623), CYP3A4 (Hs00430021), CYP3A5 (Hs01070905), and β-actin (4333762F) genes. The PCR amplifications were performed on the ABI Prism 7700 SDS (Applied Biosystems). The reactions were performed in 15 μl, using 7.5 μl of TaqMan Universal Master Mix 2×, 0.75 μl of each TaqMan probe and 1 μl of cDNA (50 ng/μl). The reactions were performed under Universal Cycling Standard Conditions (2 min at 50°C, 10 min at 95°C and 40 cycles of 15 s at 95°C, 1 min at 60°C). Each reaction was performed in triplicate, and “no template” controls were included in each experiment. The validation experiments were run to demonstrate that the efficiencies of the target and reference gene amplifications were approximately equal and within the range of 95 to 105%.

### Gene Expression Analysis

The threshold was set at 0.2 for all genes. Inter-individual variations in gene expression were calculated using version 1.6–3 software (Applied Biosystems) based on the threshold cycle (Ct) value, which defines the cycle number at which the fluorescence signal of the sample exceeds the background fluorescence value. The lowest median Ct (approximately 30 or below) correspond to genes with the highest mRNA levels and that conversely the highest median Ct (>35), thereby increasingly resulting in less robust analyses and corresponding to genes with the lowest mRNA levels. Tissue samples were considered to not be detectable and quantitative when the Ct value of the target gene was higher than 38, thereby assigning this value as “zero” in the relative expression analyses. The level of each target mRNA was examined and normalized against the β-actin gene. The ratio of copy number of the target gene over the copy number of the internal control gene was used for the relative expression level in each specimen.

### Western Analysis

Total proteins were extracted and purified by using Trizol reagent. Only 4 of 13 normal/cancer pairs were analyzed for protein expression due to insufficient material. Approximately 20 μg of protein from each sample were loaded onto a 10% SDS-polyacrylamide gel along with each of the known enzyme standards and transferred onto PVDF membranes. The membranes were blocked for 1 h at room temperature with PBS/0.05 Tween 20 containing 5% powdered milk. The membranes were then probed with primary antibodies raised against CYP1A1, CYP1A2, CYP1B1, CYP2E1, CYP2W1, CYP3A4 or CYP3A5 (Santa Cruz Biotechnology, USA) using optimized dilutions overnight at 4°C. In addition, some blots were stripped and reprobed with mouse monoclonal β-actin antibody (Sigma-Aldrich, USA) to ensure equal protein loading. Membranes were washed and treated for 1 h at 25°C with a secondary antibody (horseradish peroxidase conjugate). Protein bands were detected by enhanced chemiluminescent substrate using the Immuno-Star HRP substrate kit (Bio-Rad).

### Statistical Analysis

Descriptive statistical analysis was performed using SPSS Statistics version 17.0 (Chicago, IL, USA). The average expression levels of the CYP genes were measured with medians (25th, 75th percentiles). Differences in the expression levels between the tumor tissue and adjacent non-tumor tissue from the same individuals were evaluated using the Wilcoxon signed rank test. Associations of CYP mRNA expression levels with the clinical and histological features of tumors were measured using the appropriate statistical tests. Differences were considered to be statically significant when P<0.05.

## Results

### Characteristics of Patients and Tumors

Available clinical and histological data on all patients are summarized in Table 1. Of the 13 childhood patients with RMS diagnoses, nine were boys, and four were girls. The mean age at diagnosis was 7.9 years (range, 0.5–14.6). In our samples, the common primary sites of RMS were the head and neck (69.2%, 9/13), extremities (23%, 3/13) and genitourinary tract (7.6%, 1/13). On the basis of histological evaluation, nine tumors were categorized as the alveolar type, and four tumors were categorized as the embryonal type.

### CYP mRNA Expression in Normal and Tumor Tissue Samples

High-quality, non-degraded mRNA from normal and tumoral tissue samples was obtained from 13 patients with RMS. Expression of β-actin, which was used as a housekeeping gene, was detected in all samples. The percentage of biopsies with measurable amounts of mRNA (Ct ≤38) varied between different enzymes (Table 2). CYP1A1, CYP1A2 and CYP3A5 were detected in less than half of all the included samples, with three, four and six, respectively in cases, in compared to normal samples with four, three and six cases, respectively. In contrast, CYP2E1 mRNA was unequivocally expressed in all samples of the RMS tumor and paired non-tumor tissue from the same patients. CYP1B1 mRNA expression was detected in 12/13 normal and 11/13 paired tumor tissue samples (92% and 84%, respectively). In contrast, CYP2W1 mRNA was only detected in one non-tumor sample, obtained from a younger patient, whereas in the corresponding tumor samples, the CYP2W1 gene was expressed, and the positive rate was 61% (8/13). CYP3A4 mRNA transcripts were quantifiable in 7 out of 13 normal tissue samples (53.8%) and in 9 out of 13 tumor samples (69.2%).

**Table 2:** Expression levels of CYP genes displayed as cycle threshold (Ct) values in 13 pairs of tumor and corresponding normal adjacent tissue [Mean Ct values (mean β-actin Ct value)].

	CYP1A1				CYP1A2		CYP1B1		CYP2E1		CYP2W1		CYP3A4		CYP3A5	
	Normal		Tumor		Normal	Tumor	Normal	Tumor	Normal	Tumor	Normal	Tumor	Normal	Tumor	Normal	Tumor
Patient																
1	BLQ (26.1)		BLQ (21.2)		BLQ (26.1)	BLQ (21.2)	**34.3** (26.1)	**27.5** (21.2)	**32.2** (26.1)	**30.3** (21.2)	35.7 (26.1)	36.0 (21.2)	BLQ (26.1)	BLQ (21.2)	BLQ (26.1)	BLQ (21.2)
2	35.9 (22.9)		37.0 (21.0)		BLQ (22.9)	36.2 (21.0)	**29.3** (22.9)	**27.2** (21.0)	**31.8** (22.9)	**29.3** (21.0)	BLQ (22.9)	**32.3** (21.0)	36.5 (22.9)	**32.4** (21.0)	BLQ (22.0)	BLQ (21.0)
3	BLQ (26.0)		BLQ (32.9)		BLQ (28.2)	BLQ (32.9)	**34.1** (26.0)	BLQ (32.9)	**32.9** (28.8)	34.4 (32.9)	BLQ (32.9)	BLQ (32.9)	36.47 (26.0)	BLQ (32.9)	BLQ (26.0)	BLQ (32.9)
4	37.9 (20.8)		36.9 (19.8)		37.3 (20.8)	BLQ (19.8)	**25.5** (20.8)	**25.0** (19.8)	**27.1** (20.8)	**28.1** (19.8)	BLQ (20.8)	BLQ (19.8)	BLQ (20.8)	BLQ (19.8)	37.1 (20.8)	BLQ (19.8)
5	**34.5** (19.4)		BLQ (18.7)		37.6 (29.02)	BLQ (18.7)	**29.4** (28.1)	**24.2** (18.7)	**31.6** (34.5)	**28.4** (18.7)	BLQ (31.6)	**30.9** (18.7)	BLQ (28.1)	37.2 (18.7)	36.9 (32.4)	**34.5** (18.7)
6	BLQ (24.3)		BLQ (21.3)		BLQ (24.3)	35.2 (21.3)	**30.0** (24.3)	**25.7** (21.3)	**30.8** (24.3)	**31.4** (21.3)	BLQ (24.3)	**29.4** (21.3)	**30.8** (24.3)	**31.4** (21.3)	BLQ (24.3)	BLQ (21.3)
7	BLQ (25.5)		33.8 (23.5)		**33.2** (25.5)	**31.9** (23.5)	**26.0** (25.5)	**28.29** (23.5)	**28.2** (25.5)	**30.5** (23.5)	BLQ (25.5)	BLQ (23.5)	**28.2** (25.5)	30.5 (23.5)	**33.8** (25.5)	**33.5** (23.5)
8	BLQ (24.3)		BLQ (29.7)		BLQ (24.3)	**34.6** (29.7)	**30.39** (24.3)	**26.4** (29.7)	**34.3** (30.6)	**30.3** (29.7)	BLQ (30.6)	BLQ (29.7)	**34.3** (30.6)	**30.3** (29.7)	BLQ (24.3)	36.4 (29.7)
9	BLQ (23.9)		BLQ (22.0)		BLQ (23.9)	BLQ (22.0)	BLQ (23.9)	BLQ (22.0)	**31.8** (23.9)	**28.5** (22.0)	BLQ (23.9)	BLQ (22.0)	**31.8** (23.9)	**28.5** (22.0)	BLQ (23.9)	**32.0** (22.0)
10	37.7 (26.0)		BLQ (21.2)		BLQ (26.0)	BLQ (22.0)	**30.9** (23.9)	**27.9** (20.2)	**31.0** (23.9)	**30.1** (20.2)	BLQ (23.9)	**33.1** (20.2)	BLQ (23.9)	**30.0** (20.2)	BLQ (23.9)	**28.3** (20.2)
11	BLQ (26.2)		BLQ (19.5)		BLQ (26.1)	BLQ (21.2)	**31.9** (23.9)	**29.6** (20.2)	**32.8** (23.9)	**29.6** (20.2)	BLQ (23.9)	**34.1** (20.2)	BLQ (23.9)	36.4 (20.2)	BLQ (23.9)	BLQ (20.2)
12	BLQ (24.2)		BLQ (20.2)		BLQ (24.2)	BLQ (20.2)	**30.4** (24.2)	**26.9** (20.2)	**34.6** (24.2)	**28.9** (20.2)	BLQ (24.2)	**32.3** (20.2)	**34.6** (24.2)	**28.9** (20.2)	**34.5** (24.2)	35.7 (20.2)
13	BLQ (24.9)		BLQ (22.9)		BLQ (24.9)	BLQ (22.9)	**29.7** (24.9)	**29.3** (24.9)	**32.2** (24.9)	**32.8** (22.9)	BLQ (24.9)	35.1 (22.9)	BLQ (24.9)	BLQ (22.9)	BLQ (24.9)	BLQ (22.9)
*Mean ± standard deviation																
	36.5±1.6n = 4	35.9±1.8n = 3		36.0±2.4n = 3		34.4±1.8n = 4	29.9±2.8n = 12	27.0±1.6n = 11	31.7±2.1n = 13	30.2±1.8n = 13	35.7n = 1	32.5±2.3n = 8	32.1±3.3n = 7	31.7±3.1n = 9	35.6±1.6n = 4	33.4±2.9n = 6

BLQ: Below limit of quantification (Ct≤38); High mRNA levels (Ct ≤35) are in bold; *Mean ± standard deviation of samples with Ct ≤38 of each CYP gene; n = number of samples with Ct value ≤38. Experiments performed in triplicate.

The expression of the CYP genes in terms of Ct values (≤38) generated from RT-qPCR in tumor samples and matched normal controls are shown in Table 2. CYP genes with Ct levels of approximately 30 or below were readily detectable and quantifiable, whereas levels >35 increasingly resulted in less robust analyses. All of the sample tissues were amplified for β-actin, which was used as a housekeeping gene, with Ct value below 35. The CYP1A1 and CYP1A2 mRNA exhibited weak or undetectable expression in both normal (mean Ct>35) and tumor (mean Ct 35.9 and 34.4, respectively) tissues. In contrast, CYP1B1 and CYP2E1 were most abundantly expressed and exhibited less inter-individual variability in both normal (mean Ct of 29.9 and 31.7, respectively) and tumor (mean Ct of 27 and 30.2, correspondingly) samples. CYP2W1 in normal adjacent samples was only detected in one sample with Ct 35.7, while in the corresponding tumor samples CYP2W1 was expressed in Ct ranging from 29.4 to 36.0. Large inter-sample variability in the expression of CYP3As was found. CYP3A4 in tumor and non-tumor samples have Ct mean<35, while CYP3A5 showed Ct means of 35.6 in normal tissue and 33.4 in tumor samples.

### Expression of CYP1B1, CYP2E1, CYP2W1, CYP3A4, and CYP3A5 mRNA in Tumor and Corresponding Normal Adjacent Tissue

**Figure 1:**
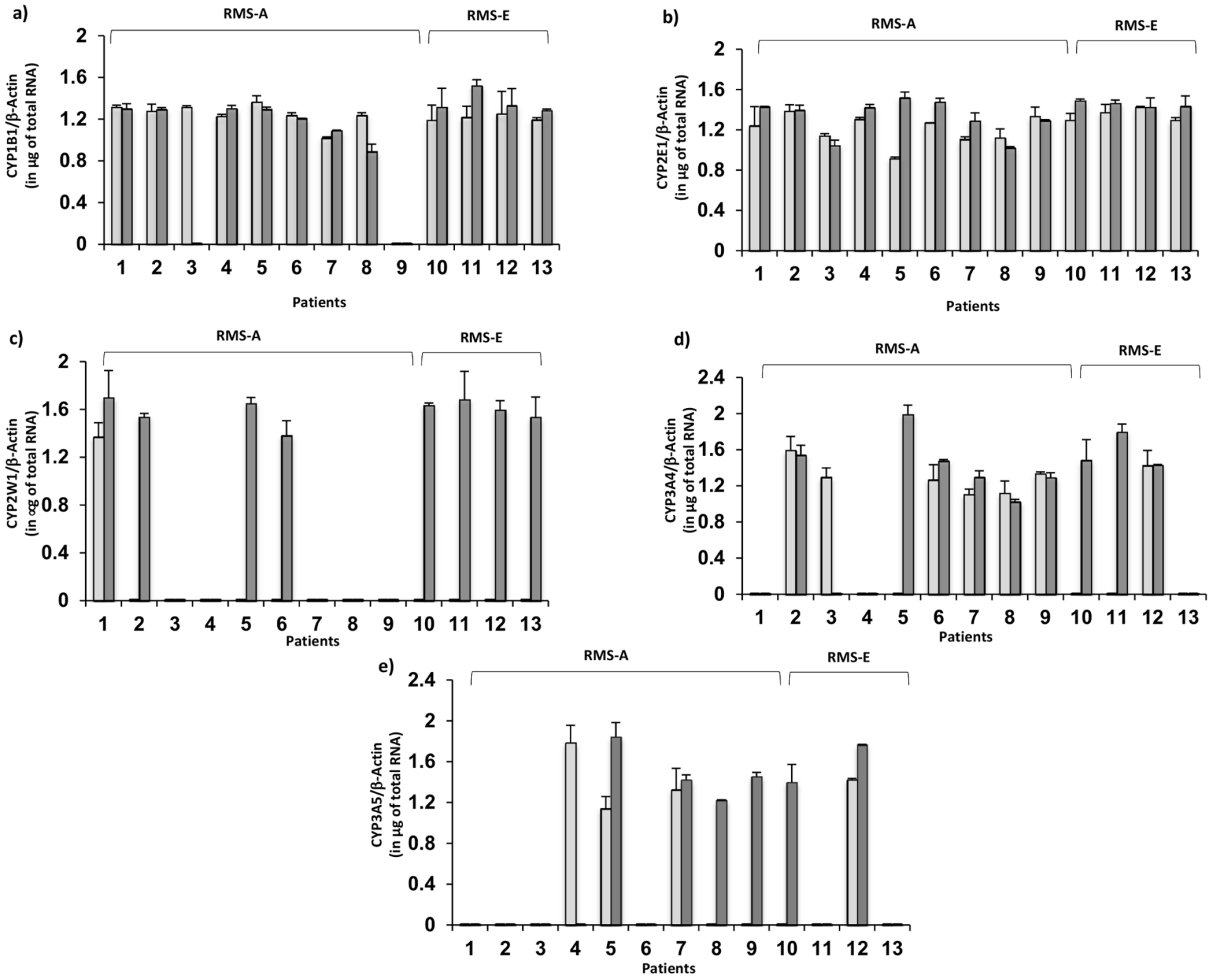
Levels of normalized mRNA Expression of (a) CYP1B1, (b) CYP2E1, (c) CYP2W1, (d) CYP3A4 and (e) CYP3A5 in 13 pairs of tumor and corresponding normal adjacent tissue from patients with RMS. Transcript levels of each CYP gene were normalized to β-actin and expressed per μg of total RNA. Values represent means and average of standard deviations of expression levels determined in triplicate. *p<0.05.

Relative expression of CYPs mRNAs in 13 matched tumor and the normal tissue pairs are shown in Figure 1. CYP1A1 and CYP1A2 genes were excluded form this comparison, since both CYPs were detected in less of the 50% of the samples analyzed (Table 2). When comparing the relative mRNA concentration of CYP1B1, CYP2E1, CYP3A4, and CYP3A5 we detected in general higher regulation in tumor than their corresponding normal adjacent samples (Figure 1). However this differential patron of expression was statistically significant in few tumor specimens. Specifically CYP1B1 (Figure 1a) and CYP3A5 (figure 1e) genes were significant upregulated in one tumor tissue (patients 4, and 7, respectively), while upregulation of CYP2E1 and CYP3A5 mRNAs were significant in 4 (patients 4, 5, 6 and 13), and 2 (patients 5 and 12) paired tumor samples, respectively (Figures 1b and Figure 1e). CYP2W1 mRNA was expressed in one matched tumor and normal tissue (patient 1), while in other 7 patients (2, 5, 6, 10, 11, 12 and 13) CYP2W1 mRNA was detected in tumor samples but not in their matched normal adjacent samples. Interestingly, we found that among the 8 tumor samples that showed CYP2W1 expression, four corresponded to embryonal type (Figure 1c).

### Interindividual Variation of CYP mRNA Expression in Normal and Tumor Tissue Samples

**Figure 2:**
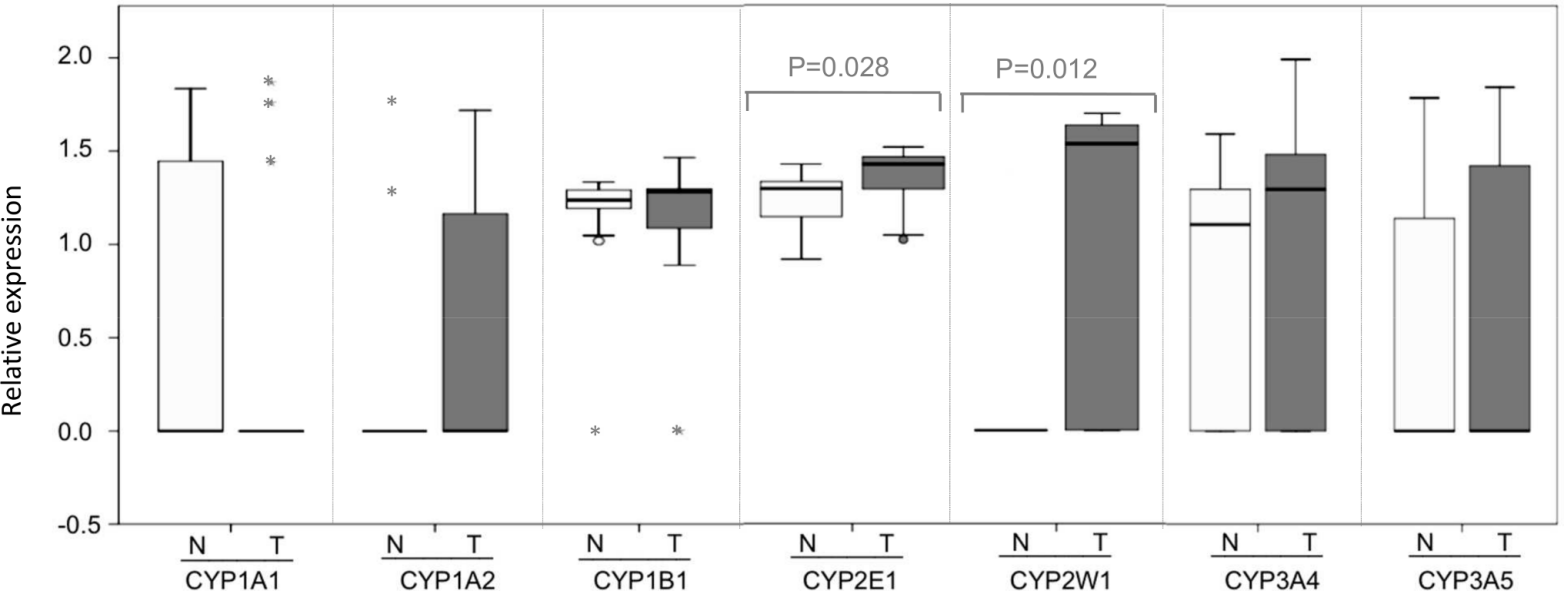
CYP expression levels in tumor and normal adjacent tissues. Box plots demonstrating the CYP-specific/β-actin mRNA ratio for each isoform in all our samples. The bottom and top of the box is the 25th and 75th percentile (the lower and upper quartiles, respectively), and the horizontal line within the box plot diagram represents the median value. The circle indicates an asterisk mark outlier values and extreme cases. The errors bars represent the range of values. Differences between tumor and adjacent normal tissue samples were tested for statistical significance using the Wilcoxon matched-pair test and are indicated with P<0.05.

Interindividual differences in the CYPs mRNAs expression levels in adjacent non-tumor tissue between tumor cancer cases were also examined (Figure 2). Data obtained from all samples, together with samples with a Ct value above 38 (assigning a numerical value of zero), were included into the relative expression analyses. CYP expression was normalized to the expression of the housekeeping gene β-actin (CYP-specific/β-actin mRNA ratio). Only CYP2E1 and CYP2W1 mRNA levels in cancer cases were significantly higher compared to normal matched samples (medians: 1.42 versus 1.29, P = 0.028, and 1.53 versus 0, P = 0.012, Wilcoxon rank-sum test, respectively). In addition, although not significant, a tendency of higher levels of CYP3A4 and CYP3A5 were found in the tumor tissue compared to the non-tumor tissue (mean: 1.02 versus 0.70, and 0.70 versus 0.43, respectively). The rest of the CYPs studied (CYP1A1, CYP1A2 and CYP1B1) showed similar mRNA relative expression levels between tumor and corresponding normal tissues.

### Correlation between CYP2E1 and CYP2W1 mRNA Values with Patient Characteristics

The interindividual expression levels of CYP2E1 and CYP2W1, as the highest expressed among the studied CYPs, were compared to the clinical and histological characteristics of the patients. As shown in Table 3, CYP2W1 overexpression in tumor RMS was significantly associated with the age of the patients (P = 0.01) but not with any other parameter. CYP2E1 expression in normal and tumor tissues did not correlate with any of the examined characteristics (P>0.05).

**Table 3:** Associations of the clinicopathological factors with CYP2E1 and CYP2W1 mRNA expression.

Variable	Tissue	CYP2E1	CYP2W1
		p-value	p-value
Age£	Non-tumor	0.36	0.10
	Tumor	0.29	0.01*
Gender£	Non-tumor	0.75	0.13
	Tumor	0.28	0.87
Histological Type§	Tumor	0.12	0.11

§ Mann-Whitney rank test.

£ Spearman rank correlation test.

*Statistically significant p<0.05.

### Protein Levels of CYPs in Normal and Tumor Tissues and their Correlation with mRNA Expression

**Figure 3:**
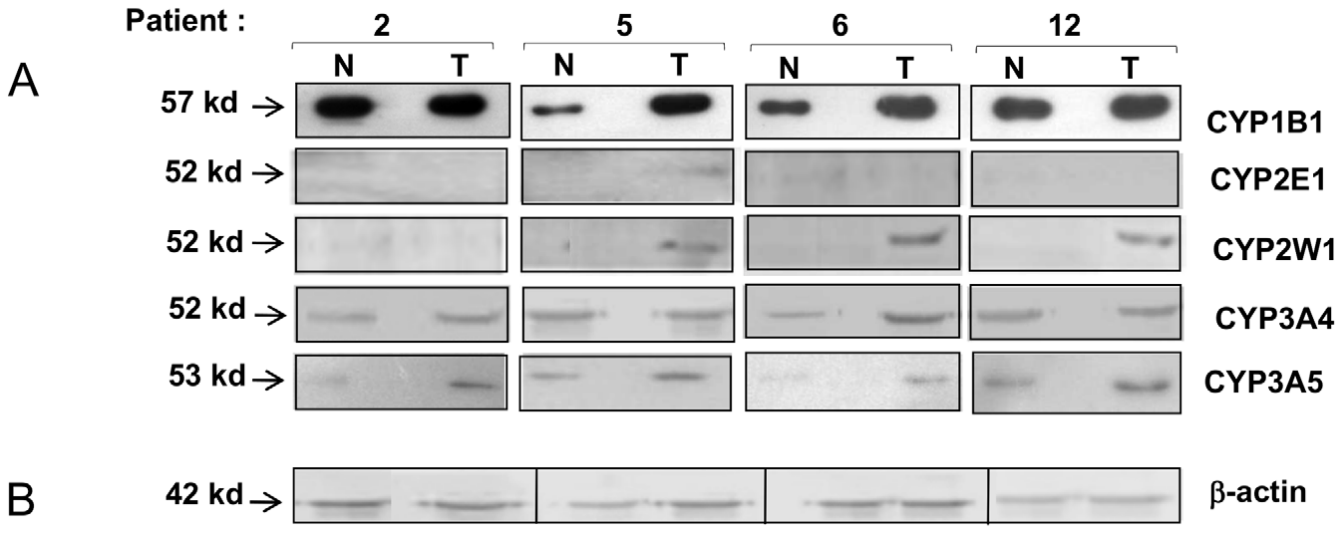
Western blots analysis of CYP proteins. (A) Representative immunoblots of CYP1B1, CYP2E1, CYP2W1, CYP3A4, and CYP3A5 proteins from non-tumor tissues (N) and tumor tissues (T) of RMS patients: 2, 5, 6, and 12. (B) The levels of β-actin were analyzed to ensure samples’ loading amount.

To further investigate whether differences found in mRNA expression of CYPs between tumor and normal tissues are related to the protein levels, protein expression of CYPs was investigated, by western blot, in four tumor and normal adjacent tissue pairs. Table 4 shows a summary of RT-PCR and Western data. Representative Western blots from selected samples are shown in Figure 3. CYP1A1 and CYP1A2 proteins could not be detected in any sample (data not shown). CYP1B1 band showed a stronger intensity in all 4 tumor and normal samples analyzed, although CYP1B1 protein was overexpressed in patients 5 and 6 in comparison to their corresponding normal tissues (Figure 3). There was a good correlation with mRNA expression (Table 4). In contrast we were able to detect abundant CYP2E1 mRNA in all normal and tumor samples by RT-PCR but not by Western blot, since CYP2E1 protein was detected only in 1 tumor sample (patient 5). Real-time PCR and western blotting analyses showed that CYP2W1 mRNA and protein expression were detected in tumor tissues but not in their matched normal adjacent samples (Table 4). In one of these tumor samples (patient 2), mRNA was detectable by RT-PCR analysis but no visible on Western blot (Figure 3). CYP3A4 and CYP3A5 proteins were variable expressed in all four pairs of tumor tissues and normal adjacent tissues since both CYPs produced higher strong bands in tumor tissues than normal adjacent tissues. These results are consistent with data from mRNA levels (Table 4).

**Table 4:** Summary of RT qPCR and Western data in four different patients.

	Patient number															
	2				5				6				12			
	N		T		N		T		N		T		N		T	
	PCR	W	PCR	W	PCR	W	PCR	W	PCR	W	PCR	W	PCR	W	PCR	W
CYP1A1	+	−	+	−	+^#^	−	−	−	+	−	−	−	−	−	−	−
CYP1A2	−	−	+	−	+	−	−	−	−	−	+^#^	−	−	−	−	−
CYP1B1	+^#^	+	+^#^	+	+^#^	+	+^#^	+	+^#^	+	+^#^	+	+^#^	+	+^#^	+
CYP2E1	+^#^	−	+^#^	−	+^#^	−	+^#^	(+)	+^#^	−	+^#^	−	+^#^	−	+^#^	−
CYP2W1	−	−	+^#^	−	−	−	+^#^	+	−	−	+^#^	+	−	−	+^#^	+
CYP3A4	+	+	+^#^	+	−	+	+	+	+^#^	(+)	+^#^	+	+^#^	+	+^#^	+
CYP3A5	−	(+)	−	+	+	+	+^#^	+	−	(+)	−	+	+^#^	+	+^#^	+

N, normal adjacent tissue; T, tumor tissue. For RT qPCR (PCR) data: −, undetectable amounts of mRNA; +, detectable mRNA expression; +^#^, high amounts of mRNA. For Western (W) analysis: −, not detected; +, positive detection; (+), weak detection.

## Discussion

The expression pattern of CYP genes affected by RMS tumorigenesis may play an important role in the progression of cancer and in the metabolism of anticancer drugs. Although the CYP mRNA levels detected in this study do not necessarily reflect active protein concentration, they can be used to predict the expression of genes into final proteins.

In the present study, we compared the pattern expression of CYP1A1, CYP1A2, CYP1B1, CYP2E1, CYP2W1, CYP3A4 and CYP3A5 in paired tumor and normal tissue of child patients with RMS.

Our motivation for analyzing these CYP genes was that CYP1A1, CYP1A2, CYP2E1 and CYP3A4, which are well-conserved, do not have important functional polymorphisms and are active in the metabolism of pre-carcinogens and drugs, while CYP1B1, CYP2W1 and CYP3A5 were more integrated because they have been determined to be overexpressed in several solid cancers [13].

In our 13 cases of RMS patients, we observed that the mean age was 7.9 years, and over 60% of the patients were less than 10 years old at diagnosis; boys (69%) were more prevalent than girls; the location sites in order of frequency were the head and neck (9/13), extremities (3/13) and genitourinary tract (1/13); finally, the majority of patients displayed the alveolar histological type (69%). Thus, the clinicopathological characteristics were consistent with the literature, with the exception of the histological type, which was most frequently the embryonal type [14].

The mRNA expression profiles of seven CYP studied in tumor and normal tissue samples were varied. In particular, genes of the CYP1 family, CYP1A1 and CYP1A2, were present at low levels in only a small number of samples (lesser 40%), while CYP1B1 was detected at high levels, mostly in tumors (Ct mean 26.9; n = 11) and normal muscle (Ct mean = 29.9; n = 12). These results were a good relation with protein expression, since we were able to detect CYP1B1 in all 4 matched tumor and the normal tissue pairs analyzed by Western blot but we don’t detected CYP1A1 and CYP1A2 proteins. These findings were consistent with previous reports, which established that constitutive expression of CYP1A1 was notably low in extrahepatic tissues [15]. Moreover, CYP1A2 protein was strictly liver-specific, indicating a very different basal regulation, although they shared induction via the aryl hydrocarbon receptor, which was similar to CYP1B1 [16].

There is abundant evidence that CYP1B1 is mainly an extrahepatic form of CYP with high basal expression in normal skeletal muscle [17], which was also observed in our study. In contrast CYP1B1 appears to be present in a wide variety of tumors [18], but we did not detect any major differences in CYP1B1 mRNA expression between non-tumor and corresponding tumor tissues. Only by Western blot we detect major CYP1B1 protein level in tumor samples than normal adjacent samples in two patients.

CYP1B1 appears to have an important role in the activation of environmental procarcinogens CYP1B1 can also biotransforms 17β-estradiol into its carcinogenic metabolite 4-hydroxyestradiol [19]. In cancer cells, CYP1B1 might induce changes in the response to drugs due to CYP1B1 activation of prodrugs, such as resveratrol, and inactivation of other drugs, such as tamoxifen and docetaxel [20], [21]. Thus, the high levels of CYP1B1 in control and malignant tissues of patients with RMS shown in this study may alter their in situ response to drugs that are substrates of CYP1B1 and may affect estrogen metabolism or activation of environmental carcinogens.

Interestingly, among the family members of CYP2 considered in this study, CYP2E1 mRNA was unequivocally expressed in all paired tumors and normal samples. CYP2W1 mRNA was detected in tumor samples (8/13) and was only slightly expressed in one sample in normal tissue. Both CYP2E1 and CYP2W1 mRNA expression in the tumor samples were higher compared to normal matched samples between patients (P<0.02 and, P<0.01, respectively). Furthermore, statistically significant associations were found between patient’s age and gene amplification for CYP2W1 in tumor samples. However to CYP2E1 no relation was found between mRNA and protein, since CYP2E1 protein was only detected in one tumor sample. In concordance with this finding in an early study has been reported a dissociation of mRNA expression and protein levels in rat colon mucosa [22]. Furthermore, using cultured hepatocytes it also has been showed that only ∼60–70% of mRNA encoding for CYP2E1 is translated [23]. Thus, the presence of CYP2E1 mRNA in normal muscle cells might have an impact on drug efficacy and toxicity as well as susceptibility to environmental toxicants, because this CYP form is important for the oxidative metabolism of several therapeutics, as well as the bioactivation of numerous toxicants (e.g., ethanol, benzene, toluene, and nitrosamines) and in the production of free radicals [24]. In this study, we demonstrated that CYP2W1 expression was upregulated at both mRNA and protein levels in several tumors compared to normal adjacent samples. Specifically CYP2W1 protein was detected in all 4-tumor samples of embryonal type RMS, suggesting that CYP2W1 is a tumor specific isoform in embryonal-RMS with potential importance as a drug target in RMS therapy; however, achieving higher confidence regarding this phenomenon would require a greater number of samples.

With respect to normal samples, CYP2W1 in mRNA only was detected in one normal tissue sample corresponding to the younger 5-month-old patient. This finding was consistent with recent findings indicating that the CYP2W1 content in human tissues gradually decreased in the first few months of life. In rat, high mRNA expression was observed in the fetal colon, with expression levels increasing with fetal age and later decreasing again after birth [25]. Similar to our findings, previous reports have determined that postnatal expression of CYP2W1 occurred only in solid tumors [26]. Previous studies have shown that the tumor-specific expression of CYP2W1 in malignant tissues was associated with a loss in epigenetic control. The exon-1/intron-1 junction of CYP2W1 gene harbors a CpG island, which is methylated in nontransformed cells and silences the gene after birth. In cancer cells, this epigenetic mark is lost, constituting a requirement for its aberrant overexpression [27].

Thus, with confirmation of the presence of protein, these patients could potentially benefit from CYP 2W1-based therapeutic interventions. In fact, the activation of an inactive prodrug by CYP2W1 generates an active product that could induce tumor- specific killing, while leaving the surrounding normal, non-CYP2W1 expressing cells unscathed. One anti-cancer agent that could be of particular interest in RMS is AQ4N, an alkylaminoanthroquinone, which is an inhibitor of both topoisomerase −1 and topoisomerase-II. This compound is activated by CYP2W1. In hypoxic conditions in tumors such as RMS, this compound produces a cytotoxic metabolite of high potency [28]. However, in normoxic conditions in normal tissues, this compound induces no cytotoxicity.

In general our analysis showed that the mRNA expression levels of the members of CYP3 family were upregulated in tumor samples in comparison their corresponding normal adjacent samples. Similar differences were found in protein levels.

Using standard inmunohistochemical methods, CYP3A4/CYP3A5 enzymes have been previously identified in various types of malignant tumors, including soft tissue sarcomas and osteosarcoma [29], [30].

The major clinical implications of putative intratumoral CYP3A4 mRNA levels were derived from the use of anticancer drugs in RMS therapy that were CYP3A4 substrates. If functionally active CYP3A4 enzymatic activity was present in RMS cancer tissue, then the enzyme could permit cancer cells to metabolize substrates, such as cyclophosphamide or ifosphamide, which are activated by CYP3A [31]. In turn, the presence of CYP3A activity would confer the ability to inactivate two widely used taxanes, such as paclitaxel and docetaxel and vinca alkaloids, in cancer cells [32]. Thus, the local expression of CYP3A enzymes in malignant tissue might contribute to the intrinsic multidrug resistance or toxicity that is observed in this type of tumor.

In conclusion, our data indicate those tumors in childhood RMS displayed higher expression of CYP2W1, CYP3A4 and CYP3A5 than did adjacent normal tissue. These findings might have implications for both the development and the treatment of this tumor. Moreover, these findings might also form the basis of a new therapeutic strategy to provide a molecular target for chemotherapeutic agents in RMS therapy. However, additional assessment of protein levels and activity measurements will be necessary to validate our results further and to confirm the translation of mRNA into functional CYP enzymes.
